# Farm or Lab? A Comparative Study of Oregano’s Leaf and Callus Volatile Isolates Chemistry and Cytotoxicity

**DOI:** 10.3390/plants12071472

**Published:** 2023-03-28

**Authors:** Antonis Kakalis, Vasileios Tsekouras, Sofia Mavrikou, Georgia Moschopoulou, Spyridon Kintzios, Epameinondas Evergetis, Vasilios Iliopoulos, Sofia D. Koulocheri, Serkos A. Haroutounian

**Affiliations:** 1 Laboratory of Cell Technology, Department of Biotechnology, Agricultural University of Athens, EU-CONEXUS European University, 11855 Athens, Greece; 2 EU-CONEXUS European University for Smart Urban Coastal Sustainability, 020276 Bucharest, Romania; 3 Laboratory of Nutritional Physiology and Feeding, Department of Animal Science, School of Animal Biosciences, Agricultural University of Athens, Iera Odos 75, 11855 Athens, Greece

**Keywords:** callus culture, cancer cytotoxicity, *Origanum vulgare*, volatile compounds, epithelial human breast cancer MDA-MB-231, human neuroblastoma SK-N-SH

## Abstract

Oregano (*Origanum vulgare*, *Lamiaceae* plant family) is a well-known aromatic herb with great commercial value, thoroughly utilized by food and pharmaceutical industries. The present work regards the comparative assessment of in vitro propagated and commercially available oregano tissue natural products. This study includes their secondary metabolites’ biosynthesis, antioxidant properties, and anticancer activities. The optimization of callus induction from derived oregano leaf explants and excessive oxidative browning was performed using various plant growth regulators, light conditions, and antioxidant compounds. The determination of oregano callus volatiles against the respective molecules in maternal herbal material was performed using gas chromatography–mass spectrometry (GC/MS) analysis. In total, the presence of twenty-seven phytochemicals was revealed in both leaf and callus extracts, from which thirteen molecules were biosynthesized in both tissues studied, seven compounds were present only in callus extracts, and seven metabolites only in leaf extracts. Carvacrol and sabinene hydrate were the prevailing volatiles in all tissues exploited, along with alkanes octacosane and triacontane and the trimethylsilyl (TMS) derivative of carvacrol that were detected in significant amounts only in callus extracts. The MTT assay was employed to assess the in vitro cytotoxic properties of oregano extracts against the epithelial human breast cancer MDA-MB-231 and the human neuroblastoma SK-N-SH cell lines. The extracts displayed concentration and time-dependent responses in cell proliferation rates.

## 1. Introduction

The demand for plant-derived secondary metabolites is growing rapidly throughout the world. A vast number of medicinal plants is collected mainly from wild populations and several species have been exhausted or even threatened due to overharvesting or habitat destruction [1,2]. Large-scale farming can provide the necessary quantity and quality of harvested biomass, while in parallel it can comply with the demands for reducing species exploitation [3,4]. However, conventional agriculture is not always advantageous since it is time-consuming and the production of desired metabolites may be inadequate due to restrictions attributed to environmental and regional conditions [5].

Plant biotechnology constitutes an alternative toolbox for the propagation of plant-derived medicinal compounds in a more effective and reliable mode [6]. Techniques, such as plant cell culture and genetic transformation, present numerous advantages for biomass generation and secondary metabolite accumulation [7]. Plant tissue culture has been widely utilized for the biosynthesis of several plant-derived metabolites such as catharanthine [8], chamazulene [9], diosgenin [10], ginsenosides [11], paclitaxel [12], rosmarinic acid [13], shikonin [14], and tropane alkaloids [15]. In vitro systems, also known as “cell factories’’, facilitate the production of desirable phytochemical compounds, manipulating metabolic pathways by shifting culture conditions such as media components, environmental parameters, growth regulators, and elicitors [9,16,17,18]. Furthermore, callus cultures are considered a sustainable and environmentally friendly method for plant biomass production, without any requirements for arable land and/or negative effects on ecosystems and biodiversity [19,20].

The production efficacy of “cell factories” has been approached and validated over naturally occurring natural products in the case of Artemisia volatiles [9]. The present study aims to address this knowledge gap, aspiring to contribute to the delineation of structure–activity relations. To achieve this, it incorporates the aromatic herb *Origanum vulgare* (*Lamiaceae*) commonly named oregano. This is a perennial shrub native to the Mediterranean region and central Asia. Oregano is one of the best-known aromatic herbs with significant commercial value, cultivated in many countries [21] and thoroughly utilized in the food and pharmaceutical industry [22,23], and its biomass propagation under controlled laboratory conditions has been described [24,25,26,27]. It is commonly used in human [28,29] and animal [30,31] diets as a spice, a source of antioxidant compounds, or as an anti-microbial agent for the in vivo inhibition of foodborne pathogens [32,33]. Different parts of the plant, either fresh or dry, display strong antioxidant properties attributed to a multitude of metabolites such as phenolic compounds, flavonoids [34], phenolic glucosides, resins, sterols, tannins, and triterpenes [22,35]. As a result, plant extracts present significant pharmaceutical properties, operating as antioxidant [36], antiviral [37], anti-inflammatory [38], cytotoxic [39], anti-neurodegenerative, and neuroprotective [40] agents. Among the diversity of oregano plant extracts outstands is essential oil, which is rich in phenols, mostly carvacrol but also thymol, and monoterpenes, such as *γ*-terpinene and *p*-cymene. These are compounds commonly utilized in the food industry as biocides, with carvacrol being recognized as therapeutic agent with antiallergic, anticancer, anticonvulsant, antidiabetic, antihypertensive, anti-inflammatory, anti-nociceptive, and antiviral properties [41,42,43,44,45].

Among this vast range of bioactivities, cancer is a major health issue and a leading cause of death worldwide, whereas projections forecast that cancer incidence will continue to grow for the next 20 years [46,47]. Conventional therapeutic modalities for cancer treatment involve combinations of surgery, radiation, and chemotherapy applications [48]. The discovery and development of novel innovative medicines are a constantly evolving field in clinical oncology [49]. Plants are the primary natural resources for the discovery of novel therapeutic compounds with pharmacological properties against cancer. Therefore, focusing on plant-derived chemotherapeutic molecules is essential, and natural compounds are widely studied against a vast number of cancer models, in vitro and in vivo [50,51]. Several phytochemicals, such as taxol analogs, vinca alkaloids, capsaicin, cucurbitacins, lycopene, and curcumin, are implicated in the growth and progression of cancer and have yielded significant results against several types of malignancies [52,53].

This context was placed in the present study’s operational framework aiming to cross-evaluate the bioactivity performance of the volatile fraction from oregano callus against the relevant isolate from the donor herbal material with respect to their phytochemical content and their bioactivity. Thus, here are the results between in vitro propagated and naturally grown oregano tissues regarding their secondary metabolite content, and their respective antioxidant and anticancer activities.

## 2. Results

### 2.1. Callus Biomass Propagation from Oregano’s Leaf Explants

#### 2.1.1. Optimizing Conditions for Callus Induction

In vitro propagated seedlings are regularly used as an alternative source of vegetative material to optimize callus generation [54,55,56]. Oregano seeds were cultured in MS (Murashige and Skoog) media and aseptic seedlings were grown to obtain leaf explants for callus induction. The cultures were carried out on an MS medium enriched by combinations of the auxin 2,4-Dichloropheoxyacetic acid (2,4-D), and the cytokinins 6-Benzyl Amino Purine (BAP) and kinetin (Kin) [24,57]. The antioxidant compound ascorbic acid was also tested as a media additive to control oxidative tissue browning, a phenomenon related to the metabolism of carbohydrates and polyphenol accumulation within cultured cells that leads to poor growth or even failure of the cultures [58,59]. It is well-documented that the photoperiod affects callus induction rate [60,61]; therefore, cultures under complete darkness and 16 h light conditions were examined.

**Figure 1:**
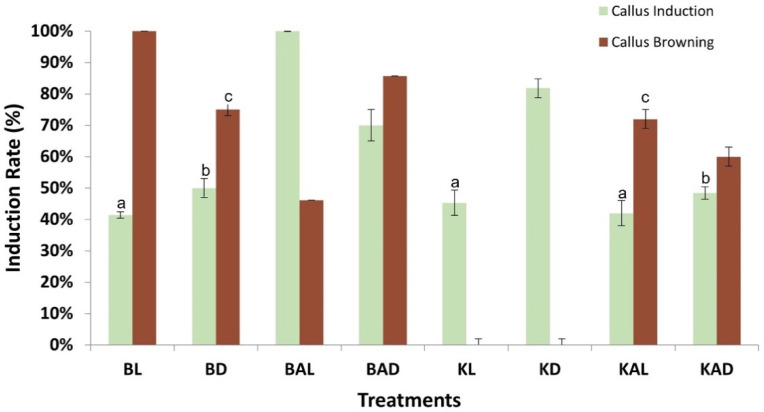
Evaluation of conditions affecting oregano callus induction from leaf explants and callus browning rate. B = MS + 0.5 mg/L 2,4 D + 3 mg/L BAP, K = MS + 2 mg/L 2,4 D + 2 mg/L Kin, A = ascorbic acid (10 mg/mL), L = 16 light, D = constant dark. Letters (a, b, c) indicate no statistical difference in callus induction among treatments.

Differential results were observed among the tested growth regulators and conditions regarding the rate of callus induction (Figure 1). Τhe combination of 0.5 mg/L 2,4-D + 3 mg/L BAP presented moderate results regarding callus induction rate reaching of 41.4% under 16/8 h light conditions (BL treatment) and 50% under dark (BD). The addition of antioxidant ascorbic acid in culture media enhances significantly the callogenesis and a maximum rate of 100% was recorded under light (BAL) and 70% in constant dark (BAD). The light conditions affected callus formation when the combination of 2 mg/L 2,4-D + 2 mg/L Kin was applied. In complete darkness (KD) the callogenesis was 81.8%, significantly higher than the 45.3% observed under light conditions (KL). The presence of ascorbic acid had a negative impact on callus induction, as the rates ranged from 42% (KAL) to 48.45% (KAD).

**Figure 2:**
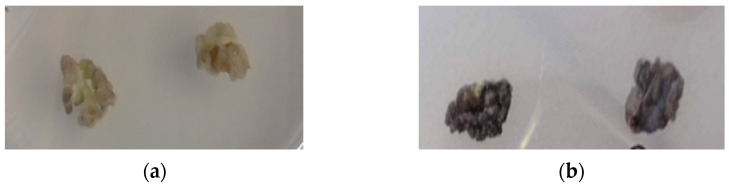
*Origanum vulgare* leaf-derived calluses. (**a**) Healthy calluses. (**b**) Brown calluses due to oxidation.

The controlling of oxidative browning is a vital parameter of tissue culture since it affects callus induction, development, and survival [62]. Culture media composition and environmental conditions significantly affect the generation of brown callus in oregano (Figure 2). Τhe application of 2,4-D and BAP displayed high rates of brown callus generation, ranging from 46.12% (BAL) to 100% (BL). On the contrary, the addition of 2,4-D and Kin, without ascorbic acid, was associated with no oxidative phenomena and all the calluses were healthy. Taken together, the inoculation of oregano leaves of MS culture media with 2 mg/L 2,4-D and 2 mg/L Kin under dark conditions (KD) results in a high induction rate (81.8%) of healthy calluses.

#### 2.1.2. Callus Biomass Production from Whole Plant-Derived Leaf Explants

The utilization of aseptic seedlings as explants’ source for tissue culture provides several advantages since the in vitro cultures are easy to handle and free of contaminants. However, whole plants are a stable, massive, and inexpensive source of explants [63]. The scope of the study is to investigate the biochemical properties of callus extracts and to identify similarities and diversities with the donor plants. Based on this, three-month-old commercial plants were used as a vegetative source for callus biomass production. Leaf explants were placed on appropriate media and culture conditions (KD) to optimize callus biomass production, as mentioned above. The plant-derived leaf explants demonstrated variances in the callus induction rate when compared to tissues harvested from in vitro propagated aseptic seedlings (Table 1).

The callus induction rate was 56.70%. Oxidative browning affected 10.91% of the propagated calluses. The average fresh weight of three-month-old calluses was 0.35 ± 0.14 gr.

### 2.2. Chemical Composition of Oregano Leaves and Leaf-Derived Calluses

**Figure 3:**
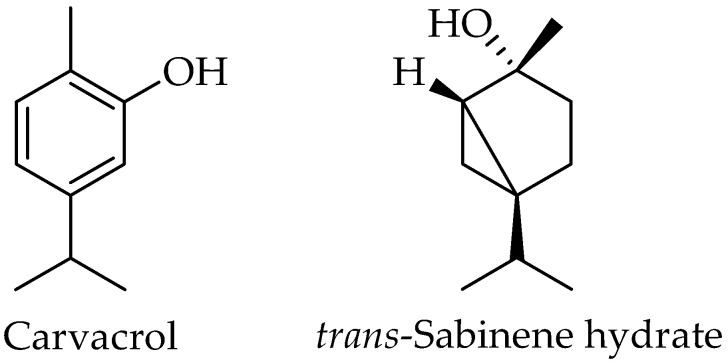
Chemical structures of carvacrol and sabinene hydrate.

In total, 27 phytochemicals were identified in both leaf and callus extracts, from which 20, accounting for 81.2%, were found in the callus extract and 20 accounted for 92% in the leaf extract. The detailed results are presented in Table 2, and the chemical structures of the two major compounds are in Figure 3.

**Figure 4:**
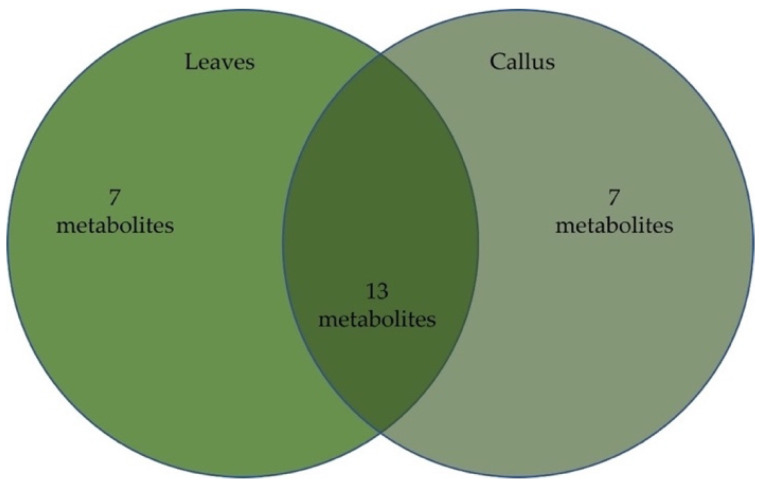
Venn diagram presenting differences and similarities in metabolite content of oregano leaves and leaf-derived calluses.

The major compounds of both extracts were carvacrol and sabinene hydrate, while in the callus extract also found as major compounds, such as the alkanes octacosane and triacontane, and the trimethylsilyl (TMS) derivative of carvacrol. Figure 4 depicts the distribution of the molecular structures within the two extracts.

### 2.3. Determination of the Antioxidant Activity by the DPPH Radical Scavenging Assay

The antioxidant activities of leaf extracts (LE) and leaf-derived callus volatile extracts (LDCE) of *O. vulgare* were evaluated using the DPPH radical scavenging assay. Their antioxidant properties were expressed as ascorbic acid equivalent (AAE) per mg of dry extract (μg/mg), calculated using the standard curve of ascorbic acid. It was observed that callus extracts showed a lower AAE than plant extracts (115.78 ± 12.22 and 259 ± 3.81 mg GAE/L, respectively).

### 2.4. Anticancer Properties of Oregano Extracts

Our study investigates the in vitro toxicity of leaf (LE) and leaf-derived callus volatile extracts (LDCE) against two cancer cell lines, the human neuroblastoma SK-N-SH and the epithelial human breast cancer MDA-MB-231 cells. The cells were treated with the extracts for 24 and 48 h and the cytotoxicity was assessed using the MTT assay.

**Figure 5:**
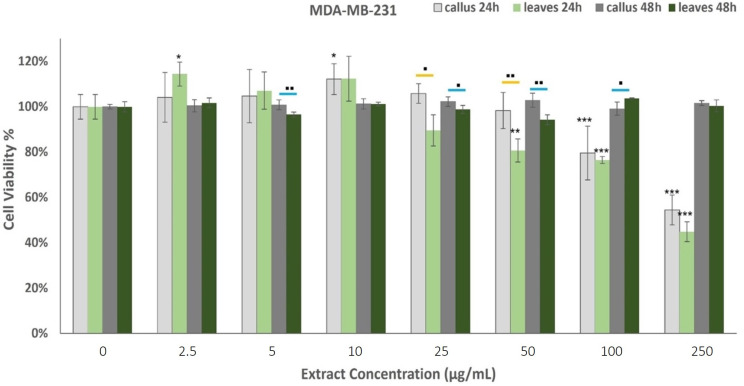
Percentage of ΜDA-ΜΒ-231 cells’ viability after treatment with the extracts derived from oregano callus and leaves at concentrations of 2.5, 5, 10, 25, 50, 100, and 250 μg/mL for 24 h and 48 h. Average results from replicate experiments ± SD (*n* = 6). The * indicates statistically significant differences from the control for the 24 h incubation, ≠ indicates statistically significant differences from the control for the 48 h incubation, and ▪ indicates statistically significant callus between callus and leaf extract when administered at the same concentration (yellow line for the 24 h incubation and blue line for the 48 h). Significant differences (Student’s *t*-test) between normalized cell responses * ▪ *p* < 0.05, ** ▪▪ *p* < 0.01, *** *p* < 0.001.

The effects of oregano extracts on MDA-MB-231 cell viability are shown in Figure 5. The results from the MTT assay indicated that the 48 h incubation with the examined extracts did not affect the cells’ viability. On the contrary, the treatment for 24 h presented dose-dependent cytotoxicity, as the callus extract (LDCE) demonstrated anticancer properties when administered at concentrations of 100 μg/mL and 250 μg/mL, while the leaf extract presented moderate effects on the proliferation rate when administered at 50 μg/mL or more. The LE was effective at lower concentrations than the LDCE but in higher doses, the observed differences were not significant.

**Figure 6:**
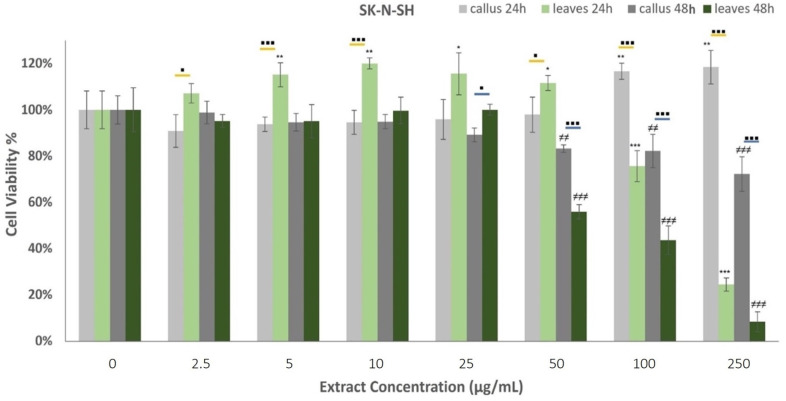
Percentage of SK-N-SH cells’ viability after treatment with extracts derived from oregano callus and leaves at concentrations of 2.5, 5, 10, 25, 50, 100, and 250 μg/mL, for 24 h and 48 h. Average results from replicate experiments ± SD (*n* = 6). The * indicates statistically significant differences from the control for the 24 h incubation, ≠ indicates statistically significant differences from the control for the 48 h incubation, and ▪ indicates statistically significant differences between callus and leaf extract when administered at the same concentration (yellow line for the 24 h incubation and blue line for the 48 h). Significant differences (Student’s *t*-test) between normalized cell responses * ▪ *p* < 0.05, ** ≠≠ *p* < 0.01, *** ≠≠≠ ▪▪▪ *p* < 0.001.

The effects of oregano extracts on SK-N-SH cell development are presented in Figure 6. All examined extracts induced concentration and time-dependent responses on cell viability. More specifically, the callus extract displayed augmented cell proliferation rates when administered at 100 μg/mL and 250 μg/mL for 24 h, whereas the leaf extract had a similarly significant influence at reduced concentrations varying from 5–50 μg/mL. Opposed to this, LE exhibited strong cytotoxic properties against the neuroblastoma cells at 100 μg/mL and 250 μg/mL treatments, demonstrating a 24.4 ± 2.9% viability rate at the highest dosage. Cells’ incubation with oregano extracts for 48 h highlighted a very strong decrease in cell viability at concentrations higher than 50 μg/mL. The findings show that the leaf extract generates stronger cytotoxic responses than the callus when applied for 48 h.

## 3. Discussion

Plant cells can generate a proliferating mass of non-differentiated cells for wounded tissue recovery, also known as a callus. Almost any plant cell can generate a callus in vitro under controlled aseptic conditions as a response to plant growth regulators and abiotic factors [64,65]. Callus generation has been previously reported for various *Origanum* species to address several demands, such as plant regeneration and secondary metabolite production in Persian (*O. vulgare* L.) and Arabian (*O. syriacum* L.) οregano [24,25], the optimization of embryogenesis and shoot regeneration of *O. vulgare* ssp. *Gracile* [66], and the assessment of antioxidant, antimicrobial, and antiviral activities of *Origanum acutidens* [67].

In our study, different plant growth regulators, light conditions, and ascorbic acid supplements were assessed for optimizing callus induction from oregano leaves. Aseptic seedlings of oregano were grown in MS media and young leaves were excised for callus induction. A maximum callogenesis rate (100%) was achieved after the inoculation of explants in media with 0.5 mg/L 2,4-D + 3 mg/L BAP, 10 mg/L of ascorbic acid and a 16/8 h photoperiod (BAL). Leaves, cultured in the same media under constant dark (BAD), displayed a reduced callogenesis rate, while the lack of the antioxidant compound in MS media significantly restrained callus formation to rates lower than 50% in both light conditions. The selection of 2 mg/L 2,4-D and 2 mg/L Kin as PGRs present a high callus induction rate of 81.8% only under constant dark (KD), whereas all the other applications failed to exceed 50% of callus generation.

Generally, the exogenous addition of auxins and cytokinins is critical for callus induction and the intermediate ratio of both PGRs in culture media is a precondition for controlling callus growth and metabolite production [68]. Leaf explants of *Origanum vulgare* L., *O.vulgare* var. *hirtum* and *O. syriacum* were cultured on an MS medium enriched with combinations of 2,4-D (2,4-Dichlorophenoxyacetic acid), Kin (kinetin), BAP (6-benzyladenine, and NAA (1-Naphthaleneacetic acid) at different concentrations. The maximum value of callus production was recorded when 0.5 mg/L of NAA and 3.0 mg/L BAP were added to the media [69]. The same culture media, fortified with BAP in combination with either NAA or 2,4-D induce the callogenesis of bud explants from *Origanum vulgare* plants growing in vitro [70]. In another study, the addition of 2.4-D in MS at low levels (up to 0.5 mg/L) was an advantageous approach for callus initiation from *O.vulgare* leaf discs, while 1 mg/L of thidiazuron (TDZ) delivered the best biomass yield during callus maintenance [24]. The combination of 3.0 mg/L Kinetin and 0.5 mg/L 2,4-dichlorophenoxy acetic, in an MS medium was beneficial for both *O. vulgare* cell suspension culture growth and poly-phenols accumulation. In this experiment, the efficacy of PGR addition in callus formation media was determined by the light conditions and the presence of antioxidant compounds. Light can regulate growth, differentiation, and metabolic processes during callus induction, influencing the levels of endogenous hormones and the efficacy of plant growth regulators. The presence or absence of light, as well as the applied wavelength, is reported to control callus formation, biomass accumulation, and metabolites biosynthesis in many plant species such as *Withania somnifera* [71], *Lemna gibba* [72], *Fagonia indica* [73], *Lavandula vera*, and *Teucrium chamaedrys* [61]. In addition, ascorbic acid is biosynthesized in plants and is involved in major metabolic activities such as photosynthesis, hormone production, cell division, and growth. During callogenesis, the enrichment of culture media with ascorbic acid displays various effects, as it is reported to improve callus induction and growth in mistletoe [74], enhance salinity tolerance in rice callus [75], and increase the biosynthesis of phenolics production in *Taxus brevifolia* [59].

Kinetin belongs to naturally occurring cytokinins, purine-based molecules that affect plant cell fate by enhancing division rates, inducing gene expression, and promoting mitosis and chloroplast occurrence [76,77]. Kinetin affects metabolic pathways, especially the phenylpropanoid pathway which is a key piece of the shikimate pathway. The pathways play a crucial role in the biosynthesis of crucial precursors for a variety of secondary metabolites necessary for plant growth such as coumarins, flavonoids, and lignans [78]. The exogenous addition of kinetin in plants via foliar applications advances crop quality, yield growth rate, and tolerance to abiotic stresses. Cytokine enhances the enzymatic and the nonenzymatic antioxidative systems of plants [79] and has been associated with the upregulation of antioxidant molecules, such as various phenolic compounds and flavonoids [76,80], as well as the activation of antioxidative enzymes such as superoxide dismutase (SOD), catalase (CAT), ascorbate peroxidase (APX), and glutathione reductase (GR) [80,81,82].

2,4-D is a synthetic auxin and is extensively used as a plant growth regulator. Exogenous 2,4-D regulates the auxin response factors (ARFs) controlling the activation of auxin response genes [83]. It is involved in auxin, abscisic acid (ABA), and ethylene biosynthesis and signaling, as well as downstream protein biosynthesis [84]. Moreover, it has been reported to promote cell division and prevent cell elongation [85]. The application of 2,4-D in callus cultures of *Caralluma tuberculate* enhances the production of phenolics and flavonoids in vitro [86]. The interplay between different hormones and their signaling pathways can modulate secondary metabolite production. For instance, the balance between auxins and cytokinins, from a moderate-high cytokinin to a low auxin ratio, has been found to affect phenolic compound accumulation in stem-derived calli from *B. pilosa* [87].

Oxidative browning occurrence is a major drawback in tissue culture that downregulates cell growth and even leads to cell death. Polyphenol oxidases catalyze the oxidation of phenolic compounds and trigger the appearance of dark pigments, posterior to mechanical damage, or oxidative stress within plant cells [88]. Callus browning is a common feature of cell cultures attributed to many factors, including species recalcitrance, physiological state, culture conditions, and media components. To alleviate the oxidation, several media supplements have been utilized, such as activated charcoal, antioxidants, enzyme inhibitors, and carbohydrates, but the phenomenon has not been surpassed yet [58,59,89]. In this report, the oxidative phenomena significantly affected the callogenesis success and, therefore, the treatment KD was selected for the establishment of leaf-derived calluses from commercially available whole plants. The results indicated that the callus induction rate was only 56.70%, mainly due to the explants’ inability to generate calluses, whereas contamination incidences were also observed. Additionally, oxidative browning appeared in 10.91% of the propagated calluses. As expected, this phenomenon was reduced under dark conditions due to diminished polyphenol biosynthesis, while treatment with 2,4-D may have promoted water absorption in proliferating cells, thus reducing the concentration of oxidized phenols. Taken together, oregano explants of different origins derived from whole plants or aseptic seedlings presented differences in callus induction rates. This can be attributed to explants’ physiological condition and up to a point, sterilization efficacy, since tissues collected from nature are less likely to be successfully decontaminated.

The phytochemical profiles of the volatile extracts obtained from callus and donor plants were proven compatible with previous results of oregano volatile studies. Specifically, carvacrol has been recognized as the dominant phytochemical in oregano’s volatile profile [90], while all other phytochemicals from the donor plant extract have been previously reported as constituents of oregano’s volatile content [90,91]. On the other hand, the callus volatile extract contained an unusually large portion of alkanes and two trimethylsilyl derivatives absent from the donor plant’s extract.

From a total of alkanes that accounted for 15.5% of the callus volatile extract, only octacosane and triacontane were detected in the donor plant extract in a much lesser percentage, just above 3%. The occurrence and quantity of these molecules may be attributed to the enhancement—during callogenesis—of the metabolism of the fatty acids, which is the biosynthetic substrate of alkane formation [92]. The TMS derivatives maybe considered analytical artifacts because of their sensitivity to water and their rapid degradation rate [93].

The literature review of the volatile extracts’ main compounds for anticancer potency revealed carvacrol to be a significantly potent molecule that has been described to present a significant antitumor effect. In colon cancer, its potential apoptotic pathway has been defined through the mitochondrial, MAPK, and PI3K/Akt signaling pathways [94]. In prostate cancer, a carvacrol dose response mode of action was confirmed [95]. Finally, the cytotoxic effects of carvacrol on human cervical cancer cells were found to hold an IC_50_ of 50 μg/mL [96]. Sabinene hydrate, on the other hand, has not attracted much research attention considering its anti-tumor activity. Specifically, sabinene hydrate as an essential oil main compound does not present significant antiproliferative activity [97]. Another relevant assessment [98] reported an IC_50_ ranging from 25 to 37 mg/mL from an essential oil that contained sabinene hydrate as the main compound. The two alkanes, octacosane, and triacontane the main compounds in callus volatile extract, have been found to display cocarcinogenic activity [99,100]; therefore, they may be considered to be cytotoxic antagonists.

Cell lines are fundamental elements of cancer research for the preclinical assessment of drug responses. In the present study, the human neuroblastoma cell line SK-N-SH and the epithelial human breast cancer cell line MDA-MB-231 were employed to evaluate the in vitro toxicity of leaf and leaf-derived callus extracts. SK-N-SH is a cancer cell line that display epithelial morphology, while MDA-MB-231 is an aggressive, highly invasive, and triple-negative breast cancer cell line that lacks an estrogen receptor, progesterone receptor, and human epidermal growth factor receptor-2 [101]. The examined extracts induced concentration and time-dependent responses in cell viability. These results adhere to the hypothesis that carvacrol is the main active compound since they exhibit positive dose-response activity. Both extracts demonstrated cytotoxic properties when administered for 24 h against MDA-MB-231 cells. The leaf extracts displayed cell proliferation reduction at lower concentrations than callus extracts, but in higher doses (100–250 μg/mL) the effects were analogous. On the contrary, the SK-N-SH cells induced the cell proliferation rate when treated for 24 h with callus extracts at high concentrations and leaf extracts at low concentrations, while the leaves exerted cytotoxic activity at concentrations higher than 100 μg/mL. This previous step for the expression of cytotoxicity (XX%) may be directly correlated with a corresponding carvacrol content of 55 and 39 μg/mL in leaf and callus volatile extracts. The decreased bioactivity of the callus extract may be attributed to the proportional decrease in the carvacrol content. Oregano extracts were highly cytotoxic when administered for 48 h against neuronal cells, whereas the leaf extracts exerted more powerful cytotoxic activities.

## 4. Materials and Methods

### 4.1. In Vitro Callus Propagation

#### 4.1.1. Plant Material and In Vitro Seedling Propagation

**Figure 7:**
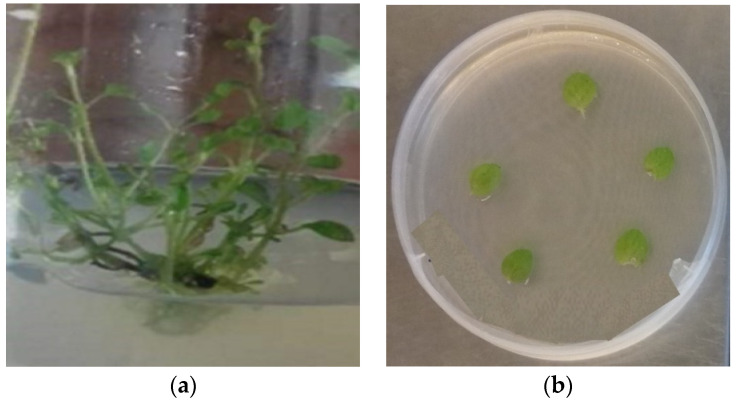
(**a**) *Origanum vulgare* seedlings growing in vitro. (**b**) *Origanum vulgare* leaves inoculated for callus induction.

Oregano (*Origanum vulgare*, *Lamiaceae*) seeds (Geniki Phytotechniki Athinon S.A., 832) were purchased from a local nursery in Athens, Greece. The seeds were thoroughly washed under tap water and then disinfected inside a laminar flow cabinet by soaking for 1 min in 70% (*v*/*v*) ethanol. Then, the seeds were treated with a solution consisting of 10% (*v*/*v*) commercial bleach and 2–3 drops of Tween-20 for 15 min and washed 3 times with sterile water. Germination was achieved after sowing in tissue culture vessels containing a Murashige and Skoog (MS) basal medium [102], supplemented with 2 mg/L of Gibberellic Acid (GA3) [24], 3% (*w*/*v*) sucrose, and solidified with 0.8% (*w*/*v*) agar. Media pH was adjusted to 5.8 and sterilized via autoclaving for 20 min at 121 °C and 1.2 atm. The cultures were inoculated in a growth chamber in dark, at 25 ± 1 °C, until germination, and the seedlings were transferred to fresh MS media, lacking GA3, at a photoperiod of 16/8 h. The plantlets (Figure 7) were subcultured in fresh media every 20 days.

#### 4.1.2. Callus Induction Optimization

Callus cultures were established after the inoculation of fresh leaves, excised from in vitro propagated two months old aseptic seedlings, on an MS medium supplemented with plant growth regulators (PGRs) such as auxin 2,4-Dichloropheoxyacetic acid (2,4-D), cytokinins 6-Benzyl Amino Purine (BAP), and kinetin (Kin) at different concentrations and combinations [24,57]. Additionally, the influence of the photoperiod and the antioxidant compound ascorbic acid on callus biomass production was assessed. The explants (*n* = 40) were placed in a plant growth chamber, at 25 ± 1 °C, under constant dark or 16 h of light, under a proton photosynthetic flux density of 50 μmol·m^−2^s^−1^. The calluses were subcultured in fresh media at 20-day intervals [9]. The different treatments applied for the optimization of callus formation are presented in Table 3.

#### 4.1.3. Leaf-Derived Calluses from Commercial Plants

Leaves were excised from commercially available plants (3 months old, *n* = 50 explants from 4 plants) of the same variety as the seeds. A part of the collected tissues was preserved at −80 °C for extraction. The leaves were disinfected following the same protocol as the seeds, and approximately 1 cm long explants were cultured on an MS medium supplemented with 2 mg/L 2,4 D and 2 mg/L Kin under constant dark. The efficacy of the sterilization processes and callus induction percentage (%) were recorded for 4 weeks, while callus fresh weight (gr) was calculated 3 months after the explants’ inoculation.

### 4.2. Herbal Tissue Extraction

Leaf-derived calluses (*n* = 20) and leaves excised from the four commercial plants (preserved at −80 °C) were dried in an oven at 40 °C and samples of the same origin were blended in one mixture. Then, the tissues were ground with mortar and pestle and the fine powder was extracted with ethanol (1:10, *w*/*v*). The mixtures were submitted to sonication for 5 min at 25 °C, and each sample was separately filtered through filter paper (Whatmann filter paper no.1). The residues were carefully collected, resuspended in ethanol, and subjected to sonication. The process was repeated three times. Finally, the extracts were combined, and the ethanol was removed by a rotary evaporator (Büchi R-210 Rotavapor System, Flawil, Switzerland) operating under reduced pressure at 30 °C. The dried leaf extracts (LE) and leaf-derived callus extracts (LDCE) were stored at −20 °C. The experimental procedures were performed in the dark to avoid interference from light.

The volatile extract was retrieved from the total ethanolic extract using the same procedure, using n-hexane as a solvent to obtain extracts for chromatographic analysis and bioassays.

### 4.3. Gas Chromatography–Mass Spectrometry (GC/MS)

All GC analyses were carried out on an Agilent Technologies 7890A gas chromatograph, fitted with an HP 5MS 30 m × 0.25 mm × 0.25 μm film thickness capillary column and FID. The column temperature was programmed from 60 to 280 °C at an initial rate of 3 °C/min. The injector and detector temperatures were programmed at 230 °C and 300 °C, respectively. Hydrogen was used as the carrier gas at a flow rate of 1 mL/min.

The GC/MS analyses were performed on the same instrument connected with an Agilent 5957C, VL MS Detector with a Triple-Axis Detector system operating in EI mode (equipped with a HP 5MS 30 m × 0.25 mm × 0.25 μm film thickness capillary column) and He as the carrier gas (1 mL/min). The initial column temperature was 60 °C and heated gradually to 280 °C at a 3 °C/min rate. The identification of the compounds was based on a comparison of their retention indices (RI) obtained, using various n-alkanes (C9–C24). Additionally, their EI-mass spectra were compared with the NIST/NBS, Wiley libraries spectra, and the literature [103,104]. Additionally, the identity of the indicated phytochemicals was confirmed by a comparison with available authentic samples.

### 4.4. Antioxidant Evaluation

Samples’ antioxidant activity was determined by the 1,1-diphenyl-2-picrylhydrazyl (DPPH) radical scavenging activity assay [105]. In brief, the DPPH solution (0.1 mM) was prepared in 90% ethanol and 50 μL of the extract (the dry material was redissolved in ethanol) was added to 1000 μL of the solution. An equal volume of ethanol (50 μL) was added to the DPPH solution as a control. The mixtures were incubated in the dark for 30 min at room temperature. The absorbance was measured at 518 nm using an ascorbic acid standard curve. The radical scavenging activity was estimated by the following equation:

%Inhibition = [(Acontrol − Asample) ÷ Acontrol] × 100

where A is the absorbance value (at 518 nm) for the control and sample.

### 4.5. Cytotoxicity Assays

#### 4.5.1. Cell Culture

Two cancer cell lines were selected, the human neuroblastoma cell line SK-N-SH (ATCC^®^ HTB-11™) and the epithelial MDA-MB-231 (ATCC^®^ HTB-26™) cells, which were grown in Dulbecco’s Modified Eagle Medium (DMEM)(Biochrom Gmbh, Berlin, Germany). The basal media was supplemented with 10% Fetal Bovine Serum (Thermo Fisher Scientific, Waltham, MA, USA), 0.5 mM sodium pyruvate, 1% penicillin, and 1% streptomycin. The cells were cultured in T-75 flasks (Sarstedt AG & Co. KG, Nümbrecht, Germany) in an appropriate incubator with humidified atmosphere at 37 °C and 5% CO_2_. Regular subcultures (twice a week) were conducted at a 1:10 ratio [106] and the adherent cells were detached from the flask by trypsinization using trypsin/ethylenediaminetetraacetic acid (EDTA) for 10 min at 37 °C.

#### 4.5.2. MTT Assay for Cell Viability

The cytotoxicity of oregano extracts (LE and LDCE) against the cancer cells was estimated with the colorimetric assay MTT (3-(4,5-dimethylthiazol-2-yl)-2,5-diphenyltetrazolium bromide) (Duchefa Biochemie, Haarlem, The Netherlands) [107]. Specifically, transparent flat-bottom 96-well plates (SPL Life Sciences Co., Ltd., Naechon-Myeon, Republic of Korea) were seeded with 100 μL of cell suspension with a density of 8 × 10^3^ cells and 6 × 10^3^ cells for 24 h and 48 h of incubation, respectively. The plate was placed in the chamber culture for 24 h and then the culture medium was removed and substituted with 200 μL of DMEM with 1% FBS and various concentrations of the extracts (2.5, 5, 10, 25, 50, 100, and 250 μg/mL). Dimethyl sulfoxide (DMSO) was utilized for the re-solubilization of the dry extracts. Cells treated with culture media only were the control (0) of the assay. The MTT dye was added to the cells at a concentration of 0.5 mg/mL. After a three-hour treatment with the MTT, the culture medium was removed and 200 μL of dimethyl sulfoxide (DMSO) was added for cell lysis. The cells’ viability was estimated by recording changes in absorbance at a wavelength of 560 nm (PowerWave 240 Microplate photometer, Biotek, Winooski, VT, USA).

### 4.6. Statistical Analysis

The obtained data were analyzed using SPSS v. 16.0 package software. Parametric data were statistically analyzed by one-way analysis of variance (ANOVA) and Student’s *t*-tests for pairs. *p*-values < 0.05 were considered to be statistically significant. The assays were performed in duplicates, with 6 technical replicates.

## 5. Conclusions

In conclusion, the present study advocated the potential of aromatic plants as a promising target for cell factory development. The presented results indicated that oregano biotechnological biosynthesis concludes that extracts are comparable in composition and bioactivity with the ones that are naturally produced. Although the in vitro culture technique is a promising alternative for the production of natural compounds, further exploration is required to elucidate the target compound’s biosynthetic mechanisms in order to maximize their cell factory yield. In this study, it was indicated that the application of streamlined analytical techniques and bioassays may produce valuable insights for the valorization and alleviation of known and well-established crop pharmaceutical potentials.

## Figures and Tables

**Table 1:** Callogenesis rate of leaf explants derived from whole plants.

Infected Explants	Callus Induction	Brown Callus	Healthy Callus
8.49%	56.70%	10.91%	46.23%

**Table 2:** Volatile extract analyses. Compounds are listed in order of elution from an HP-5MS column (RI = Kovats Indices calculated against C8 to C24 n-alkanes on the HP-5MS column). a = RI comparison, b = MS comparison, c = authentic sample comparison. nd: not detected.

Components	RI	Leaves	Callus	Identification
*β*-Thujene	910	0.2	nd	a, b
*α*-phellandrene	915	nd	0.2	a, b
Sabinene	962	0.4	nd	a, b, c
*β*-Pinene	964	nd	0.3	a, b
*β*-Myrcene	986	0.6	0.7	a, b
*α*-Terpinene	1012	0.4	0.2	a, b
*o*-Cymene	1020	1.2	0.7	a, b
*β*-Phellandrene	1024	0.5	nd	a, b, c
*γ*-Terpinene	1055	1.9	0.4	a, b
*cis*-Sabinenehydrate	1062	1.7	0.8	a, b
*trans*-Sabinenehydrate	1094	13.9	7.5	a, b
Linalool	1100	3.0	nd	a, b, c
Borneol	1161	0.3	nd	a, b
Terpinen-4-ol	1174	0.3	1.8	a, b, c
Terpineol	1187	1.9	1.3	a, b, c
Linalyl acetate	1256	4.4	nd	a, b
Thymol	1295	0.4	0.1	a, b, c
Carvacrol	1304	55.4	39.2	a, b, c
Carvvacrol-1-TMS	1334	nd	9.4	a, b
Caryophyllene	1415	1.3	1.5	a, b, c
Bicyclogermacrene	1495	0.7	nd	a, b
1-Hexadecanol-1-TMS	1964	nd	0.9	a, b
Hexadodecanoic acid ethylester	1992	nd	0.7	a, b
Hexacosane	3088	nd	2.1	a, b, c
Octacosane	3279	1.6	6.5	a, b, c
Nonacosane	3485	nd	0.7	a, b, c
Triacontane	3543	2.1	6.2	a, b, c
**Total**		**92.0**	**81.2**	

**Table 3:** Different concentrations of PGRs and ascorbic acid in culture media and culture conditions for the optimization of callus induction.

Treatment Code	2,4 D (mg/L)	Cytokine (mg/L)		Ascorbic Acid (mg/L)	Light (Hours)
		BAP	Kinetin		
BL	0.5	3	-	-	16
BD	0.5	3	-	-	-
BAL	0.5	3	-	10	16
BAD	0.5	3	-	10	-
KL	2	-	2	-	16
KD	2	-	2	-	-
KAL	2	-	2	10	16
KAD	2	-	2	10	-

## Data Availability

The data are contained within the article.
